# Prevalence and associated risk factors for *Giardia* and *Cryptosporidium* infections among children of northwest Mexico: a cross-sectional study

**DOI:** 10.1186/s12889-017-4822-6

**Published:** 2017-10-30

**Authors:** Luis Quihui-Cota, Gloria Guadalupe Morales-Figueroa, Aarón Javalera-Duarte, José Antonio Ponce-Martínez, Edith Valbuena-Gregorio, Marco Antonio López-Mata

**Affiliations:** 1 Departamento de Nutrición Pública y Salud, Coordinación de Nutrición, Centro de Investigación en Alimentación y Desarrollo, A.C. Hermosillo, Sonora México; 2 Departamento de Ciencias de la Salud, Universidad de Sonora, Campus Cajeme, Cd. Obregón, Sonora México

**Keywords:** Giardiasis, Cryptosporidiosis, Children, Northwest Mexico

## Abstract

**Background:**

*G. intestinalis* and *Cryptosporidium* spp. are responsible for gastrointestinal infections worldwide. Contaminated food, feces, drinking water and predictors such as poverty, cultural and behavioral aspects have been involved in their transmission. Published studies about these infections are limited in Mexico. Cananea, Sonora is located in northwest Mexico and is one of the regions with the lowest marginalization index in the Sonora state. However, its rate of gastrointestinal infections increased from 48.7/1000 in 2003 to 77.9/1000 in 2010 in the general population. It was estimated that the prevalence of giardiasis can range from 20 to 30% in the Sonoran childhood population. However, the prevalence of giardiasis and cryptosporidiosis are unknown in Cananea, Sonora and they are likely contributing to its gastrointestinal infections rates.

**Methods:**

A total of 173 children (average age 8.8 ± 2.8 years) participated in this cross-sectional study. Anthropometric measurements and stool analysis were performed. Socioeconomic, cultural and symptomatology information were collected. The association between the risk factors and intestinal parasitic infections was analyzed by multivariate analysis using the STATA/SE package at a significance level of *p* ≤ 0.05.

**Results:**

More than half of the children (*n* = 103, 60%) had intestinal parasitic infections. *Cryptosporidium* spp. showed the highest prevalence (*n* = 47, 27%), which was followed by *G. intestinalis* (*n* = 40, 23%). Children with giardiasis and cryptosporidiosis had lower H/A and BMI/A Z scores than children who were free of these infections. Children with giardiasis were at higher risk (OR = 4.0; 95%CI = 1.11–13.02; *p* = 0.030) of reporting abdominal pain, and children who drank tap water were at higher risk (OR = 5.0; 95% CI = 1.41–17.20; *p* = 0.012) of cryptosporidiosis.

**Conclusions:**

This was the first epidemiological study conducted in children in the region of Cananea, Sonora in northwest Mexico. The findings revealed a high prevalence of cryptosporidiosis and giardiasis, and their interactions with multiple risk factors were investigated. This study suggested that giardiasis and cryptosporidiosis may play an important role as causative factors of gastrointestinal diseases in the study region. Regional authorities must analyze water for human consumption to search for *Cryptosporidium* spp. and *G. intestinalis*.

## Background

It was estimated that approximately two hundred million people were affected yearly with giardiasis in Africa, Asia and Latin America [1]. In addition, the overall prevalence of giardiasis was estimated from 2 to 5% in industrialized countries [2]; meanwhile, the prevalence of *Giardia intestinalis* (*G. intestinalis*) in developing countries was estimated as 15 to 20% in children younger than 10 years of age. Another protozoan, *Cryptosporidium* spp., is found worldwide with a prevalence ranging from 4 to 31% in some developing countries [3]. *C. parvum* and *G. intestinalis* have been recognized as important causes of gastrointestinal infections and diarrhea [4], and they can be transmitted by person to person via fecal-oral route or indirect route through contaminated food and drinking water with human or animal feces. For the above reasons, additional factors contributing to the spread of the parasitic infections are socioeconomic status, age, household crowding, education, animal ownership and lack of access to clean water and proper sanitation [5]. In Mexico, the prevalence of giardiasis can range from 3% up to 50% in different regions [6], but the epidemiological trends of cryptosporidiosis remain unknown at the national level. In 2006, sporadic published information highlighted that 41% of 100 infants hospitalized had *Cryptosporidium* spp. in a public health institution located in Mexico city [7]. In 2013, Olivas-Enriquez et al. detected *C. parvum* in 19 of 38 (50%) household water samples and in 12 of 13 study communities [8]. In 2010, it was published that 5.1% of 5459 infants from 1 month to 5 years old had *Cryptosporidium parvum* in Guadalajara, Mexico [9]. Otherwise, it was estimated that 14% (372,742 people) of 2,662,480 inhabitants in the state of Sonora (northwest Mexico) lived in rural communities [10]. Sonora is the second largest Mexican state based on territorial area, and it consists of 72 regions and Cananea is one of them. Some years ago a health report pointed out that Cananea had the second highest rate of gastrointestinal infections (increased from 48.7/1000 in 2003 to 77.9/1000 in 2010) at the state level [11]. Based on this, giardiasis and cryptosporidiosis may be associated with a high rate of gastrointestinal infections reported in that region. Because these infections remain unknown in the region of Cananea, we investigated the prevalence levels of giardiasis and cryptosporidiosis and their association with socioeconomic factors and behavioral habits in children from that region. The information will be useful for preventing and controlling these infections by the regional health authorities.

## Methods

### Study site and population

**Fig. 1:**
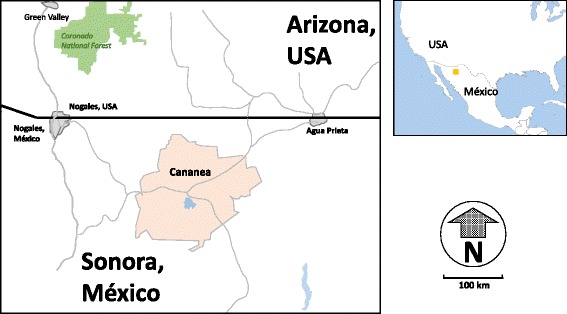
Region of Cananea, Sonora at northwest Mexico

This was a cross-sectional study conducted from February 2013 to September 2014 in the region of Cananea, Sonora (northwest Mexico). Cananea has a population of 32,936 inhabitants [10]; it is located at 1654 m above sea level and is bordered to the north by the United States and other Sonoran municipalities such as Naco to the northwest, Arizpe to the south, and Imuris and Santa Cruz to the west (Fig. 1). Cananea has a humid warm climate with an average annual temperature of 15.3 °C, average temperatures of 18 °C and 14 °C during spring and autumn, respectively, and annual rainfall of 545 mm. For this study, three kindergarten and two public primary schools in the region of Cananea were selected because they were located in low income level areas [12] and had high rates of gastrointestinal diseases. A total of 366 preschoolers and 491 school children (total *n* = 857) who were officially enrolled in the selected schools (school year 2013–2014) [13] were invited to participate. At the same time plastic containers were distributed to collect stool samples (three per child). The study protocol was explained to the school authorities and parents.

### Ethical consideration

Informed consent that explained the purposes, benefits, and risks of the study was provided to each parent or guardian of the participating children prior to starting the study. Two hundred and three children wished to participate in this study. One hundred and seventy-three (20%) children fulfilled the required study criteria. The parents of 30 (3.7%) children did not sign the informed consent. The remaining children (*n* = 654, 76.3%) did not participate for various reasons. Both participating and non-participating children lived in the same living conditions around the selected kindergartens and schools. The ethics committee of the Centro de Investigación en Alimentación y Desarrollo approved this study. Children with intestinal parasitic infections received the proper treatment from an experienced physician.

### Anthropometric measurements

Standing height was measured using a stadiometer (Holtain Ltd., Dyfed, UK) with 2.05 ± 0.001 m capacity, and weight was measured to the nearest 10 g using a digital electronic scale (AND FV-150 KA1, A&D Co. Ltd., Toshima-ku, Tokyo, Japan) according to the standardized recommendations. Ages were validated from reliable official school records. The weight-for-age (W/A), height-for-age (H/A), and body mass index-for-age (BMI/A) Z scores were calculated using the WHO Anthro for personal computers, version 1.0.4, 2009: Software for assessing the growth and development of the world’s children [14]. Undernutrition risk was defined from −2 to < −1 Z scores and moderate and severe undernutrition from ˂ -2 Z scores considering the median reference values of H/A (stunting), W/A, and BMI/A [15].

### Fecal sample collection, processing, and analysis

In the Faust technique [16], each fecal sample was poured into a round-bottom tube (100 by 13 mm) to within 20 mm of the rim. Then, 3 mL of distilled water was added, and the fecal material and water were mixed. The suspension was centrifuged for 10 min at 2500 rpm (700 × g). All centrifugations were performed without mechanical breaking. The supernatant was decanted, and the last drop was drained onto a clean section of paper towel. This washing procedure was repeated 3 times. Then, 3.5 ml of aqueous ZnSO_3_ solution (1.180 specific gravity) was added to within 50.8 mm of the rim of the tube. The packed sediment was re-suspended using applicator sticks until no coarse particles remained. This suspension was centrifuged for 5 min at 2500 rpm (700 × g) and transferred without agitation to a rack that held it upright; the suspension was then allowed to stand for 20 min. With a wire loop of 5 mm in diameter bent at a right angle to the stem, two loops of the surface film were transferred to a drop of iodine solution (Weigert’s solution) on a glass slide (76.2 by 50.8 mm) for wet-mount examination. Then, 10× and 40× objectives were used for identification of cysts of *G. intestinalis*. The specific gravity of the zinc sulfate solution was checked every 7 days using a calibrated hydrometer with a specific gravity range of 1.00 to 1.20 throughout the study. In addition, 1 g of fecal sample was smeared on clean slides and stained with the cold acid-fast Kinyoun stain [17] to identify *Cryptosporidium* spp. oocysts by light microscopy at a magnification of ×100.

### Collection of information from children and their families

The collection of particular and socioeconomic information in this study was performed with a structured and locally adapted questionnaire [18]. The interviews were administered face-to-face with parents in the children’s households. A well-trained technician and the person responsible for the study conducted the interviews to lessen potential bias. Several scoring indices were constructed to describe the population. The civil status of the parents was assessed from the married status and was assigned (0) for synonymous with stability or (1) for risk or low stability. The socioeconomic status was assessed from the employment status and parental education, assigning (0) for employed or (1) for unemployed, (0) for complete or (1) for incomplete secondary school. Household conditions were assessed by the type of material used for walls (brick, adobe, block or cardboard), roofs (cement or aluminum plus wood) and floors (ground, cement or mosaic), which were categorized based on local costs of materials and the presence (0) or absence (1) of window nets. Sanitation facilities and hygiene indices were assessed as the use of a flushing toilet (0) or a latrine (1); drinking water was assessed as treated water (0) or tap water (1). We inquired about proper hygiene and hand-washing before eating, after restroom use, or after touching pets present in the household, and these were assigned (0) if “always” or (1) if “not always” and food washing as (0) if “always” or (1) if “not always”. Crowding was estimated using the number of people per room, and it was categorized as less than three (0) or more than three people (1) per room in agreement with the WHO 2006 guidelines [19]. Family income (including economic support from different sources) was estimated as the number of minimum daily-wages ($67.3 pesos or $4.86 USD with the valid type of exchange of $13.84 pesos per dollar at the study time) [20, 21] from dividing the daily family income by the current local minimum daily wage. The health status of the children was investigated based on the visible child’s symptomatology or present symptoms in the last 7 days given by the mothers at the time of interview. The values of (0) and (1) were assigned for the absence and presence, respectively, of abdominal pain and headache. Finally, the absence (0) or presence (1) of domestic animals in the households was investigated.

### Statistical analysis

An exploratory analysis of data from the database was conducted. Age and anthropometric indicators of the study children were expressed as a mean value with standard deviation. The prevalence of infection or parasite species was expressed as the percentage of children with pathogenic or commensal protozoa or each identified spp. or genera of protozoa present in any of the provided fecal samples. Analysis of covariance (ANCOVA) examined the differences between mean values taking into account the influence of the uncontrolled independent variables and Wilcoxon rank-sum test were used to test the differences between medians. The proportions were compared using the chi-square test with the corresponding odds ratios (OR), 95% confidence intervals and two-sided *p* values. The association between the risk factors and intestinal parasitic infections was analyzed using both univariate analysis by simple logistic regression and multiple logistic regression analysis. All plausible biological variables with OR > 1 and *p* ≤ 0.2 in univariate analysis were selected for multiple logistic regression analysis using stepwise forward elimination with an acceptance criterion of *p* ≤ 0.05 and the adjusted OR. The resulting preliminary model was evaluated by interaction (*p* ≤ 0.1) and collinearity (correlation coefficient > 0.7) to generate the final adjusted model.

In all constructed models, the dependent variable was intestinal parasitic infection and the risk factor was the hypothesized independent variable. The variables judged to be possible confounding factors, such as sex and Z scores for the anthropometric indices, were used in the multiple logistic regression analysis. Regression diagnostics to identify outliers and influential data points were also conducted. All data were analyzed using statistical software STATA/SE version 12.0 (StataCorp. 2011. Stata Statistical Software: Release 12. College Station, TX: StataCorp LP).

## Results

### Prevalence of *Giardia* and *Cryptosporidium*

A total of 488 fecal samples were collected and transported in ice boxes to the parasitology laboratory of the Centro de Investigación en Alimentación y Desarrollo and were stored between 5 °C and 7 °C for 24–72 h until analysis. Eighty-seven percent (*n* = 150), 8% (*n* = 15) and 5% (*n* = 8) of the participant children gave 3, 2 and 1 sample(s) per child, respectively.

The overall prevalence of pathogenic and commensal protozoa found in the participant children (*n* = 173) is shown in Table 1. More than half (*n* = 103) of the children had protozoan infections (60%), and 29% (*n* = 50) had infections with two or more protozoan genera. No difference was found in the prevalence of protozoan infections between females and males (*p* = 0.290; data not shown). *Cryptosporidium* spp. showed the highest prevalence (*n* = 47, 27%), which was followed by *G. intestinalis.* (*n* = 40, 23%). In addition, *E. histolytica/dispar/moshkovskii* were also detected, albeit at a lower prevalence. However, the species of the *E. histolytica/dispar/moshkovskii* complex were not identified. On the other hand, *E. nana* had the highest prevalence (*n* = 57, 33%) of all pathogenic and commensal protozoa detected in this study. *I. bütschlii* had a lower prevalence (*n* = 1, 0.6%). Finally, the helminth *H. nana* was only found in 4 children (2%) (not shown in Table 1). *E. histolytica* is well recognized as a pathogenic amoeba, but *E. dispar*, *E. nana*, *E. coli* and *I. bütschlii* are considered a non-pathogenic amoebae [22]. However, pathogenicity of *E. moschkovskii* remains unknown [23].

**Table 1:** Prevalence of pathogenic and commensal protozoa in 173 children of the region of Cananea in northwest Mexico

Status (n)^a^	Percentage (95% CI)^d^
Uninfected (70)	40 (33–48)
Infection^b^ (103)	60 (52–67)
Polyparasitism^c^(50)	29 (22–36)
Pathogenics	
*Cryptosporidium* spp. (47)	27 (20–33)
*Giardia intestinalis* (40)	23 (17–29)
*Entamoeba histolytica/dispar/moshkovskii* ^e^ (4)	2 (0.5–4.5)
Commensal	
*Endolimax nana* (57)	33 (26–40)
*Entamoeba coli* (29)	17 (11–23)
*Iodamoeba bütschlii* (1)	0.6 (0.02–1.7)

^a^Number of cases

^b^Infection with one or more genera or species (cases of *H. nana* included)

^c^Infection with two or more genera or species (cases of *H. nana* included)

^d^Confidence interval

^e^Pathogenic/non-pathogenic protozoa

### Infection and nutritional status

At baseline, the average age of the study children (*n* = 173) was 8.8 (±2.8) years old. Fifty-one percent (*n* = 88) and 49% (*n* = 85) were female and male, respectively. No difference was found between the proportions of female and male participants (*p* = 0.747). Our study children consisted of 26 preschools and 147 primary school children with average ages of 3.6 (±0.98) and 9.7 (±1.93), respectively. In relation to the anthropometric measurements, the proportions below −2 SD in W/A, H/A (stunting) and BMI/A were 1.5% (*n* = 3), 3.3% (*n* = 6) and 3% (*n* = 5), respectively. The means (SD) for the W/A, H/A and BMI/A Z scores were 0.135 (±1.12), −0.53 (±1.41) and 0.42 (±1.40), respectively.

No difference was found in the age, weight, height, W/A Z scores between the children with and without giardiasis or cryptosporidiosis (Table 2). However, children with giardiasis or cryptosporidiosis had significantly lower H/A and BMI/A Z scores than uninfected children (*p* = 0.001 and *p* = 0.028 and *p* = 002 and *p* = 0.030 respectively) (Table 2).

**Table 2:** Characteristics of the free and infected children with *Giardia* and *Cryptosporidium* of the region of Cananea in northwest Mexico

Variable	Free of pathogenic species	*G. intestinalis infected	*p* value^e^
	*n* = 93	*n* = 40	
Age (years)	8.5 (±2.9)	8.7 (±2.9)	0.716^c^
Weight (kg)^a^	21.6 (18.2–23.9)	25.6 (20.8–31.6)	0.379^b^
Height (cm)	125.2 (±24.3)	123.5 (±19.2)	0.667^c^
W/A (Z score)^d^	0.31 (±1.1) (*n* = 65)	−0.14 (±1.1) (*n* = 25)	0.089^c^
H/A (Z score)	−0.11 (±1.6)	−1.05 (±1.0)	0.001^c^
BMI/A (Z score)	0.67 (±1.3)^b^	0.06 (±1.5)	0.028^c^
	Free of pathogenic species	**Cryptosporidium spp. infected	
	*n* = 93	*n* = 47	
Age (years)	8.5 (±2.9)	9.2 (±2.8)	0.170^c^
Weight (kg)^a^	21.6 (18.2–23.9)	29.1 (23.2–36.7)	0.201^b^
Height (cm)	125.2 (±24.3)	124.3 (13.1)	0.776^c^
W/A (Z score)^d^	0.31 (±1.1) (*n* = 65)	−0.09 (±1.1) (*n* = 35)	0.087^c^
H/A (Z score)	−0.11 (±1.6)	−0.83 (±1.1)	0.002^c^
BMI/A (Z score)	0.67 (±1.32)^b^	0.13 (±1.4)	0.030^c^

*Children with *G. intestinalis* and other pathogenic and commensal species

**Children with *Cryptosporidium spp*. and other pathogenic and commensal species

^a^Median (25–75% inter-quantiles); remaining data are presented as mean ± standard deviation

^b^Wilcoxon rank-sum test

^c^ANCOVA adjusted by other pathogenic genera or species rather than *G. intestinalis or **Cryptosporidium spp.

^d^Not all W/A Z scores of the children could be estimated

### Distribution of risk factors of the families of participating children

One hundred and seventy-three mothers and 45 fathers of the children responded to the questionnaires. Most of the participating children came from homes with unmarried mothers and fathers (Table 3). At the time of the interview, most mothers were housewives and most fathers were employed. Most parents had not completed their secondary education, and more than half of the families were living with 1 or less valid minimum wages. Twenty-four percent of the families of the participating children were living in crowded conditions, and 90% were living in households made of materials that were appropriate for the weather conditions of the study area. Also, most of the children’s families drank water directly from the tap (59%). In addition, 43% of the families had domestic animals (Table 3).

**Table 3:** Distribution of risk factors collected from parents of participant children of the region of Cananea in northwest Mexico

Characteristic	Number of interviews	Categories	Percentage (n)
Civil status			
Mother	173	Married	32 (56)
		Unmarried (free union, widow, single)	68 (117)
Father	45^a^	Married	30 (14)
		Unmarried (free union)	70 (31)
Economic activity			
Mother	173	Yes (sales, teacher, babysitter, adult patient care, cleaning of homes, maid, laundry, merchant, employee, retired)	43 (74)
		No (housewife, student)	57
Father	45	Yes (sowing, mechanic, laborer, sales, miner, merchant, painter, teacher, blacksmith, carpenter, welder)	88 (40)
		No (student)	12
Education			
Mother	173	Completed secondary	25 (40)
		Uncompleted secondary	75
Father	45	Completed secondary	25 (11)
		Uncompleted secondary	75
Family Income	173		
		˃ 4.86 USD	21 (36)
		≤4.86 USD (≤1 minimum wage/day)	79
Crowding	173		
		<3 persons per room	76 (131)
		>3 persons per room	24
Household characteristics	173		
Walls material		Cement, brick, block, adobe	90 (156)
		Other material (cardboard)	10
Floor material		Laminate, mosaic, cement	90 (156)
		Ground	10
Roof material		Cement, wood and metal laminate	90 (156)
		Only metal laminate	10
Drinking Water	173		
		Treated water (boiled or with iodine and/or chlorine solution or commercial purified water)	41 (71)
		Direct tap water (only with chlorine 5 ppm).	59
Symptomatology	173		
Abdominal pain			
		Yes	64 (111)
		No	36
Headache			
		Yes	46 (80)
		No	54
Domestic animals	173		
		No	57 (99)
		Yes (chickens, dogs, cats)	43

^a^Present as family member confirmed by the mother at the time of the interview. The rest of the families had no paternal figure

### Factors associated with *G. intestinalis* and *Cryptosporidium* spp.

*G. intestinalis* and *Cryptosporidium* spp. were separately analyzed as dependent variables and risk factors as independent variables. Univariate analysis revealed that the drinking water type, presence of domestic animals in the household, symptomatology and seasonality for sample collection for giardiasis; and drinking water type and presence of domestic animals in the household for cryptosporidiosis fulfilled the acceptance criterion for examination in the multiple regression analysis (Table 4). The civil status, education and economic activity of the parents; family income; crowding and household conditions did not meet the criteria (data not shown). The nutritional status, sex and age are recognized to be factors that influence the prevalence of intestinal parasitic infections [24, 25]. They were used as adjustment variables in the multiple regression logistic analysis (stepwise), and preliminary models for giardiasis and cryptosporidiosis were produced. Interaction (*p* > 0.1) and collinearity (*p* < 0.7) were not found for those models, and the final models were defined (Table 5). The *Giardia*-infected children had higher risk to present abdominal pain (OR = 4.0, 95%CI = 1.11–13.02, *p* = 0.030) adjusted by sex, age, W/A, H/A, and BMI/A Z-scores. On the other hand, children drinking tap water were at a higher risk (OR = 5.0, 95%CI =1.41–17.20, *p* = 0.012) of cryptosporidiosis with the same adjustment variables (Table 5).

**Table 4:** Univariate analysis of giardiasis and cryptosporidiosis with risk factors in 173 children from three kindergarten and two public primary schools of the region of Cananea, Sonora in northwest Mexico

	*Giardia*				*Cryptosporidium*			
Variable	n	*Giardia* infected^a^	OR (95%CI)	p*	n	*Cryptosporidium* spp. infected^b^	OR (95%CI)	p*
Drinking water	40				47			
Treated water		9	1.0			12	1.0	
Tap water		31	3.9 (1.7–8.9)	0.014		35	3.9 (1.64–9.10)	0.002
Keep domestic animals	40				47			
No		23	1.0			20	1.0	
Yes		17	2.8 (1.30–5.88)	0.008		27	3.5 (1.55–7.56)	0.002
Symptoms	40				47			
No Abdominal pain		23	1.0			16	1.0	
Abdominal pain		17	3.2 (1.32–7.81)	0.010		31	1.54 (0.69–3.45)	0.290
No Headache		23	1.0			27	1.0	
Headache		17	2.9 (1.35–6.02)	0.006		20	0.74 (0.35–1.51)	0.390
Season for sample collection	40				42			
Autumn		8	1.0			16	1.0	
Spring		32	3.53 (1.45–8.56)	0.005		26	1.1 (0.50–2.25)	0.902

OR (95%CI) = Odds ratio (95% confidence interval)

*p significant at ≤0.05 (logistic regression analysis)

^a^Children with *G. intestinalis* and other pathogenic and commensal genera or species

^b^Children with *Cryptosporidium spp*. and other pathogenic and commensal genera or species

**Table 5:** Multiple logistic regression analysis of giardiasis and cryptosporidiosis with the risk factors that fulfilled the acceptance criterion in the univariate analysis

*Giardia* infected^a^ (*n* = 40)	OR	(95%CI)	*p
Abdominal pain	4.00	1.11–13.02	0.030
Sex^c^	1.81	0.67–4.90	0.237
Age^c^	0.84	0.65–1.05	0.178
W/A (Z score)^c^	2.81	0.98–8.09	0.054
H/A (Z score)^c^	0.30	0.15–0.64	0.002
BMI/A (Z score)^c^	0.13	0.18–0.92	0.032
*Cryptosporidium* spp. infected^b^ (*n* = 47)	OR	(95%CI)	*p
Tap water	5.00	1.41–17.20	0.012
Sex^c^	0.47	0.15–1.40	0.190
Age^c^	1.56	1.11–2.18	0.010
W/A (Z score)^c^	3.88	1.23–12.20	0.020
H/A (Z score)^c^	0.39	0.20–0.77	0.007
BMI/A (Z score)^c^	0.35	0.14–0.85	0.210

OR (95%CI) = Odds ratio (95% confidence interval)

*p significant at ≤0.05 (Multiple logistic regression analysis)

^a^Children with *G. intestinalis* and other pathogenic and commensal genera or species

^b^Children with *Cryptosporidium spp*. and other pathogenic and commensal genera or species

^c^Adjustment variables

## Discussion

This study concerned the prevalence of *G. intestinalis* and *Crypstosporidium* spp*.* and the risk factors associated with their presence. The study was performed from February 2013 to September 2014 in children from two public elementary schools and three public kindergartens located in the region of Cananea in northwestern Mexico. More than half of the children (*n* = 103, 60%) had infections by pathogenic and commensal protozoa, and one-third had intestinal polyparasitism (*n* = 50, 29%). In 2010, Sánchez and Miramontes [26] also found that nearly half (42.2%) of 2055 children from 3 to 19 years of age in 14 rural communities in San Luis Potosí had intestinal parasites. In addition, no difference was found in the prevalence of these infections between boys and girls or school and preschool children in this study. Some years ago, another local study found no difference (*p* = 0.989) in the prevalence of these infections between male (*n* = 157) and female (*n* = 155) school children of different rural and suburban areas in southern Sonora. These children are probably exposed to the same risk factors, irrespective of their sex [27]. In addition, a recent systematic epidemiological review (*n* = 103 studies) in Iran revealed that the similar prevalences of these infections between Iranian preschool and school children (38.2 and 43.4%, respectively) are probably influenced by factors such as sanitation, hygiene, awareness of people, seasonal variations and health education [28]. On the other hand, *G. intestinalis* (*n* = 40, 23%) remains an important protozoan that causes infection in school children in northwest México [29]. Conversely, Sánchez and Miramontes found a low prevalence of giardiasis (approximately 5%) in 2126 children from 3 to 19 years of age [26], which is probably associated with the massive antiparasitic campaigns in their study communities. In this study, the prevalence of cryptosporidiosis (*n* = 47, 27%) was as high as giardiasis (23%). Therefore, this study revealed that *G. intestinalis* is not the only predominant protozoan affecting children in Cananea in northwest Mexico, but it is also accompanied by *Cryptosporidium* spp. Otherwise, the helminth *H. nana* and the protozoa *E. histolytica/dispar/moshkovskii* were found to have low prevalences (2 and 2.3%, respectively). These prevalence levels were lower than those published in 2015 for these organisms (16 and 10%, respectively) in school children from different communities of the Sonora state [27]. The low prevalence of *H. nana* is probably associated with the national albendazole campaign that is administered twice a year in the study site [29], but the low prevalence of *E. histolytica/dispar/moshkovskii* is probably a result of the poor efficacy of the Faust technique for detecting these organisms [30, 31]. Sanchez and Miramontes [26] observed the same finding (approximately 1.2% for *E. histolytica*) using the Faust technique. It should be remarked that these prevalence data may be underestimated because proper molecular assays are more effective and sensitive than microscopic methods for the detection of parasitic infections and differentiation of species. However, the high cost is still a limiting factor. On the other hand, a high prevalence of commensal protozoa was found in our study (*E. nana*, 33% and *E. coli*, 17%). These protozoa are indicators of poor sanitary conditions, and they are a public health concern because they use the same transmission routes as pathogenic organisms [32]. With respect to the nutritional status, the study children had a prevalence of undernutrition according to H/A (stunting) and W/A Z scores that were lower than and similar to, respectively, the prevalence published by the national survey in 2012 (3.3% vs. 13.6% and 1.5% vs. 1.6%) [33]. However, it should be emphazised that our study children are not representative of the entire population of the Sonora state.

On the other hand, children with giardiasis and cryptosporidiosis had lower H/A and BMI/A Z scores than children free of these infections (Table 2) which is probably a result of the parasite interfering with intestinal absorption leading to malnutrition. Duran et al., [34] found in 3388 children that the average BMI of the *Giardia* infected children was significantly lower than in those free of these infection (17.86 ± 0.22 kg/m2 versus 19.49 ± 0.097 kg/m2). Similarly, a study in Brazil found a lower H/A Z score in children with giardiasis than in *Giardia*-free children in 2007 [35]. These authors suggested that giardiasis can reduce the weight of affected children, but chronic infection may contribute to the height deficits observed in their study communities. In our study, giardiasis or cryptosporidiosis was associated with stunting, and different studies have shown that even asymptomatic giardiasis and cryptosporidiosis can be associated with growth shortfalls [36, 37]. However, it should be emphasized that factors such as re-infection, chronic infection and chronic poor dietary intake can potentially predispose children to stunting.

Furthermore, the system of potable water is of great concern to the transmission of intestinal protozoan infections in the study site. Drinking water directly from tap was a risk factor of cryptosporidiosis (OR = 4.42) in the participating children. The agency responsible of water management at local level add 5.0 mg of chlorine per litter of water to an area of concentrating water before is distributed to the water distribution network of the Cananea region. However, *Cryptosporidium* spp. is resistant to conventional disinfectants and it has been reported active infections in mice inoculated with 60,000 oocysts exposed for 90 min with 80 mg of chlorine per litter of water [38]. On a worldwide scale, there is great concern for authorities responsible for providing safe drinking water for human consumption because an increasing number of waterborne outbreaks of this infection have been reported, particularly in the USA and UK. Innovative technologies to improve detection, monitoring and surveillance of *Cryptosporidium* appeared after 1996, which is when the American Federal Government considered these to be drinking water contaminants and prompted the USEPA to create and implement drinking water regulations [4]. On the other hand, there are drinking water regulations in Mexico with standards that do not include *Cryptosporidium* spp. analysis [39]. No association was found between giardiasis and cryptosporidiosis with the age of the children, the civil status and education of the parents, family income, crowding, household conditions, domestic animals and seasonality or domestic animals at home (data not shown). On the other hand, symptomatology was associated with giardiasis, but not with cryptosporidiosis. Probably this resulted of the reflection of children’s susceptibility to giardiasis or cryptosporidiosis in this study [3]. Finally, this was the first epidemiological study in Cananea focusing on children. Our study has limitations to be considered while interpreting the results. We conducted three stool examinations to detect *G. intestinalis* and *Cryptosporidium* spp., but 8% (*n* = 14) and 5% (*n* = 9) of the children gave two and one sample(s), respectively. Because optimal laboratory diagnosis of parasites in stool samples requires the examination of at least three specimens collected over different days, some degree of underestimation could be present. Previous studies have suggested that one stool sample has a sensitivity of between 50 and 70%, but three serial samples have a sensitivity of up to 90% [40]. Conclusions about the causality of associations between different factors and the detected protozoa could not be drawn because this is a cross-sectional study design. Most socioeconomic characteristics of the participating children were not associated with giardiasis or cryptosporidiosis in this study, which is probably related to the low sample size. On the other, although this study had a low sample size, it was sufficiently strong to identify a significant association between giardiasis and cryptosporidiosis and several variables even after adjusting for the presence of other factors in the analysis.

## Conclusion

This study provided data on the prevalence and important data regarding risk factors for giardiasis and cryptosporidiosis. These infections are a concern of public health in the studied children of the region of Cananea, Sonora, northwest Mexico. The data suggested that giardiasis and cryptsoporidiosis may contribute to height deficits in our study children. On the other hand, because domestic tap water is a risk indicator of infection, monitoring bacteria in drinking water, as dictated by the Mexican law, should be accompanied by analysis of *G. intestinalis* and *Cryptosporidium* to assess the quality standards of drinking water in the study site. It was also suggested that giardiasis and cryptosporidiosis may be somewhat responsible for the rate of gastrointestinal diseases present in the population of that region. Based on these findings, actions must be taken by the regional health authorities to reduce and prevent gastrointestinal infections as well as to monitor the quality of the drinking water in the study site.
